# Supplementary material for: FragGeneScanRs: faster gene prediction for short reads
Source: BMC Bioinformatics. 2022 May 28;23:198. doi: 10.1186/s12859-022-04736-5 (PMC9148508; doi:10.1186/s12859-022-04736-5)
Supplement: Supplementary file 1 — Additional file 1. Examples of input problematic for FGS+. A PDF file describing in detail some examples of input problematic for FGS+ [file 12859_2022_4736_MOESM1_ESM.pdf]

Examples of input problematic for FGS+

Incorrect translation of reverse strand

```
>AIL77220.1 Rieske (2Fe-2S) protein [Acinetobacter baumannii] 136527..136832
TGTTCTGCTTCCTTGTACATGTGAGGACTAGAGTTGAATTTGAATATCCTGTCCTTCAACACGAACTGGGTACGTACATA
GGTTTCTACACCCGGGTCCACTTTTTAGCTCACCTGTCTGAATGTCAAAAATTGCTTCGTGCAATGGACATTCCACAGTT
TGATCTTCGATAAATCCTTCCGTTAATAAGGCATACGCATGAGGGCAAACATTTTCGATAGCGAAGTAATTTTCATCGAC
AAAAAAAACACCAATTTTTTTCCCTTCAACTTCGACGGCTTTCGGCTCATCTTCGCTAACGTCACCCTGCTGACAAACTG
AGATCCAACTCATACCTTGCGTCCTCATTTTGTTTTATATACAAAACATAATTTGATTTTCAAAACACAAGCTAAGCATA
ATCCTCTTGATTAATTTTTGTCAAAGTAAAAATAAACATTAAAATCAATTGATTAATAAATTTTAAATAATTTGTTACGT
TTCAAGTCAGAAACAATGTTTTAAATATAAAAATTGTTTTATGTAATCTTTATAATTACAATAGTTCTAAA
```

Expected predicted amino acid sequence:

```
MRTQGMSWISVCQQGDVSEDEPKAVEVEGKKIGVFFVDENYFAIENVCPHAYALLTEGFIEDQTVECPLHEAIFDIQTGE
LKSGPGCRNLCTYPVRVEGQDIQIQL
```

FGS+'s predicted amino acid sequence:

```
$ ./FGS+ -s /tmp/test_fasta.txt -o stdout -w 1 -t illumina_10
ELNLNILSFNTNWVRTVSTPGSTFLTCLNVKNCFVQWTFHSLIFDKSFR**GIRMRANIFDSEVIFIDKKNTNFFPFNFD
GFRLIFANVTLLTN*DPTHTLRPH
```

(reported by our team at https://github.com/hallamlab/FragGeneScanPlus/issues/19)

Ignored `-e` and `-d` options

FGS+ ignores the presence of the `-e 1` and `-d 1` flags, rendering it unable to generate the metadata and DNA files. Example invocation:

```
FGS+ -s test-contigs.fasta -o test -w 1 -t complete -p 24 -m 30000 -e 1 -d 1
```

Expectation: `test.ffn` and `test.out` should be created.

(reported in https://github.com/hallamlab/FragGeneScanPlus/issues/10)

FGS+ hangs indefinitely on short standard input

Using the same short file from above, FGS+ (sometimes) hangs indefinitely on the input.

```
$ ./FGS+ -s stdin -o stdout -w 1 -t illumina_10 < /tmp/test_fasta.txt
Max memory limit specified invalid, defaulting to 1024MB
```

(reported by our team at https://github.com/hallamlab/FragGeneScanPlus/issues/6)

FGS+ slows down disproportionately on very long reads

Most easily tested with a complete genome as ‘read’, e.g. https://www.ebi.ac.uk/ena/browser/view/GCA_001628815?show=chromosomes.

```
$ ./FGS+ -s ena_data_20210917-1328.fasta -o stdout -w 0 -t complete
Max memory limit specified invalid, defaulting to 1024MB
```
