# Supplementary material for: FragGeneScanRs: faster gene prediction for short reads
Source: BMC Bioinformatics. 2022 May 28;23:198. doi: 10.1186/s12859-022-04736-5 (PMC9148508; doi:10.1186/s12859-022-04736-5)
Supplement: Supplementary file 2 — Additional file 2. Performance on simulated reads. Detailed benchmark results on a collection of simulated data sets [file 12859_2022_4736_MOESM2_ESM.pdf]

Performance on simulated reads

Benchmarks

To better demonstrate the difference in processing speed, a collection of data sets was simulated using Mason (Holtgrewe M, 2010). Each dataset contains 10K illumina reads of varying read length simulated from Geobacter anodireducens strain:SD-1 (ENA Project PRJNA315482, sequences CP014963.1 and CP014964.1). The commands used were:

```
# for read length <l> from 100 to 1000
mason_simulator -ir ena_data_20210917-1328.fasta -n 10000 --illumina-read-length <l> \
  --fragment-mean-size 2000 --fragment-min-size 1000 -o <l>.fasta -oa <l>.sam

# for read length <l> from 2000 to 10000
mason_simulator -ir ena_data_20210917-1328.fasta -n 10000 --illumina-read-length <l> \
  --fragment-mean-size 20000 --fragment-min-size 1000 -o <l>.fasta -oa <l>.sam
```

These commands result in reads with following properties (more default properties may be found in the Mason documentation):

- 0.00005 insert per-base probability for insertion.
- 0.00005 insert per-base probability for deletion.
- 0.004 average per-base mismatch probability.
- 0.002 per-base mismatch probability of first base.
- 0.012 per-base mismatch probability of last base.

The commands used to measure execution time were:

```
FragGeneScan -s <l>.fasta -o FGS<l> -t illumina_10 -w 0
FGS+ -s <l>.fasta -o FGS<l> -t illumina_10 -w 0
FGSrs -s <l>.fasta -a FGS<l>.faa -t illumina_10 -w 0
```

Predictor	Read length	Mean [s]	Min [s]	Max [s]
FragGeneScan	100	4.843 ± 0.018	4.815	4.873
FGS+	100	0.491 ± 0.002	0.489	0.495
FragGeneScanRs	100	0.396 ± 0.004	0.394	0.405
FragGeneScan	200	5.966 ± 0.028	5.939	6.039
FGS+	200	0.842 ± 0.001	0.840	0.843
FragGeneScanRs	200	0.748 ± 0.002	0.745	0.750
FragGeneScan	300	7.020 ± 0.011	6.999	7.038
FGS+	300	1.168 ± 0.001	1.166	1.171
FragGeneScanRs	300	1.103 ± 0.012	1.097	1.135
FragGeneScan	400	8.073 ± 0.020	8.050	8.116
FGS+	400	1.487 ± 0.002	1.485	1.493
FragGeneScanRs	400	1.451 ± 0.002	1.448	1.453
FragGeneScan	500	9.109 ± 0.016	9.083	9.141
FGS+	500	1.803 ± 0.004	1.799	1.813
FragGeneScanRs	500	1.795 ± 0.002	1.792	1.799
FragGeneScan	600	10.122 ± 0.021	10.101	10.175
FGS+	600	2.115 ± 0.002	2.111	2.117
FragGeneScanRs	600	2.154 ± 0.015	2.141	2.191
FragGeneScan	700	11.137 ± 0.026	11.110	11.189
FGS+	700	2.448 ± 0.007	2.441	2.467
FragGeneScanRs	700	2.505 ± 0.008	2.491	2.517
FragGeneScan	800	12.196 ± 0.020	12.174	12.238
FGS+	800	2.790 ± 0.005	2.780	2.795
FragGeneScanRs	800	2.859 ± 0.010	2.851	2.879
FragGeneScan	900	13.218 ± 0.023	13.177	13.252
FGS+	900	3.132 ± 0.003	3.129	3.140
FragGeneScanRs	900	3.198 ± 0.010	3.191	3.225
FragGeneScan	1000	14.309 ± 0.255	14.175	15.014
FGS+	1000	3.474 ± 0.018	3.462	3.526
FragGeneScanRs	1000	3.547 ± 0.005	3.539	3.556

Predictor	Read length	Mean [s]	Min [s]	Max [s]
FragGeneScan	2000	24.443 ± 0.025	24.413	24.497
FGS+	2000	7.021 ± 0.020	7.006	7.069
FragGeneScanRs	2000	7.052 ± 0.024	7.035	7.118
FragGeneScan	3000	34.655 ± 0.038	34.602	34.705
FGS+	3000	10.913 ± 0.014	10.888	10.943
FragGeneScanRs	3000	10.545 ± 0.025	10.514	10.594
FragGeneScan	4000	45.037 ± 0.051	44.969	45.111
FGS+	4000	14.841 ± 0.020	14.805	14.872
FragGeneScanRs	4000	14.042 ± 0.022	13.996	14.070
FragGeneScan	5000	55.315 ± 0.059	55.223	55.430
FGS+	5000	18.756 ± 0.034	18.714	18.813
FragGeneScanRs	5000	17.525 ± 0.039	17.465	17.605
FragGeneScan	6000	65.537 ± 0.118	65.414	65.714
FGS+	6000	22.997 ± 0.298	22.814	23.835
FragGeneScanRs	6000	21.007 ± 0.028	20.974	21.061
FragGeneScan	7000	75.791 ± 0.058	75.732	75.892
FGS+	7000	27.099 ± 0.080	26.998	27.256
FragGeneScanRs	7000	24.521 ± 0.068	24.443	24.680
FragGeneScan	8000	86.172 ± 0.202	86.053	86.715
FGS+	8000	31.440 ± 0.051	31.391	31.539
FragGeneScanRs	8000	27.980 ± 0.043	27.915	28.047
FragGeneScan	9000	96.354 ± 0.347	96.145	97.329
FGS+	9000	35.858 ± 0.040	35.795	35.940
FragGeneScanRs	9000	31.489 ± 0.055	31.409	31.580
FragGeneScan	10000	106.509 ± 0.104	106.411	106.764
FGS+	10000	40.513 ± 0.071	40.439	40.635
FragGeneScanRs	10000	34.962 ± 0.050	34.861	35.033

Predictions

The simulated read dataset of 10K reads with an average length of 2000 base pairs was also used to evaluate the predictions made. For each simulated read, the annotated genes on the reference sequence were compared to the predictions made by each tool. A base pair is considered positive if it lies withing a predicted gene; true positive (TP) if it also lies withing an annotated gene on the same strand and false positive (FP) if it does not lie within an annotated gene or on the other strand. A base pair is considered negative if it does not lie withing a predicted gene; true negative (TN) if it also does not lie within an annotated gene and false negative (FN) otherwise.

The python script performing these measures can be found in the meta directory of the GitHub repository.

predictor	TP	FP	TN	FN	prec	sens	spec	NPV	MCC
FGS	63.59%	28.80%	4.63%	2.98%	68.83%	95.52%	13.86%	60.85%	0.17
FGSrs	63.59%	28.79%	4.64%	2.98%	68.84%	95.52%	13.88%	60.89%	0.17

The minor difference between FGS and FGSrs is caused by a difference in rounding method in C and Rust. For completeness, the diverging predictions were studied using the GNU `diff` tool. They fall into these categories:

Category	Frequency
FGSrs predicts a more than 10 bp shorter fragment	6
The prediction start and end differ less than 10 bp	37
FGSrs predicts a more than 10 bp longer fragment	8
FGSrs predicts multiple close gene fragments rather than a single long one	4
FGSrs predicts a single long gene fragments rather than a multiple short ones	1
FGSrs leaves out a prediction	2
